# Loot

**Published:** 1873-11

**Authors:** 


### Loot.

### *Normal Ovariotomy.*

The new operation of normal ovariotomy is engaging the thought and comment of the profession so generally at the present time as to make everything bearing upon the subject of interest to our readers.

It affords us pleasure to be able to put upon record some additional facts, derived from a second case operated upon by Prof. J. T. Gilmore, of Mobile, Alabama, and kindly furnished us in the subjoined letter from that skilled surgeon:

MOBILE, ALA., June 15, 1873.

*Dear Doctor*—Your letter relative to a case of normal ovariotomy was received some days ago.

The case I operated upon was unlike yours, in having an atrophied uterus of many years duration. Whilst the menstrual irritation had, since the menopause, been productive of great discomfort, it was only within the six months immediately preceding the operation that it had threatened life by the violent congestions of the brain which were recurring regularly every two weeks at the period of the operation, at which time she had become almost idiotic.

She was in bad plight for any operative procedure at the time it was done, and yet, so threatening were the paroxysms, it was feared that another one would destroy her life.

Drs. Ketchum and Gaines had attended her for years and had resorted to every resource in the effort to re-establish the menstrual flow, but without avail, and they both assented to the proposition that if normal ovariotomy was a legitimate operation under any circumstances, the case in question justly claimed its benefits. Dr. Ketchum assented to the operation. Dr. Gaines hesitated, but his objection presented no real basis upon which to ground an opposition. Drs. W. H. Anderson, Ketchum and Gaines discussed the probable physiological effect of a removal of the ovaries, and all agreed that without some such intervention the patient would certainly not get through with many more such attacks as she had recently endured.

Dr. Anderson, who fills the Chair of Physiology in our medical college, urged the operation upon physiological grounds. The absence of the uterus rendered it improbable that there could ever be any more menstrual elimination, and the purpose of the operation would be to destroy, as far as possible, the menstrual irritation, by putting a stop to ovulation.

The operation was performed in December last.

The peritonitis that was lighted up, and the cellulitis which followed, ended in abscess, which is still discharging through the abdominal wall, at the point of incision. The patient barely escaped with her life.

Her brain is now relieved. Every two weeks she has still some hysterical symptoms, *i. e.*, choking sensations, with the feeling as though there were some foreign body in the throat. There has been no menstrual show whatever. There is still discharging from the abdominal fistula about an ounce and a half of thin pus daily, and the cavity of the abscess I wash out every third or fourth day with carbolized water, through a catheter and syringe. The abdominal discharge is lessening daily, and there are noticeable changes in her features and mammary glands.

I shall await the full results of the case before publishing it in detail. This is an important subject you have opened up to the profession, and deserves a most honest and careful detail of cases operated. Yours truly, J. T. GILMORE.

We hope, at a proper time in the future, to be able to lay before our readers the full particulars of this case, the operation and its results, both physiological and curative.—*Atlanta Med. and Surg. Jour.*

### *Fracture of the Clavicle Treated by Placing the Arm behind the Back.*

A patient was recently under M. Broca's care who had fractured, by a fall, his left clavicle, near the middle of the bone. The fracture was oblique, from above downwards and from without inwards, the fragments riding over one another to a considerable extent. Various plans of treatment were tried, but without effecting permanent reduction into a good position. At length, calling to mind a communication made last year by Dr. Michel, to the Societe de Chirurgie, M. Broca placed the limb in a semi-flexed position behind the back, when the most perfect confluence of the fragments occurred. The arm was fixed in this position by a bandage, and kept in it for eighteen days. At the expiration of this time the bandage was removed and the arm set at liberty. When it was found that the parts were sufficiently consolidated to prevent any likelihood of displacement, the limb was brought forward and kept immovable in a sling for a few days longer. This method of treatment has been regarded as excessively painful, but in this instance the patient only complained of the inconvenience and pain for the first twenty-four hours. At the same time it must be stated that he was a man of considerable nerve. Immediately after his entrance into the hospital, he several times raised his hand to his head, giving a fresh demonstration of the possibility of movement with a fractured clavicle. The result was so good, says M. Broca, that had the patient been a lady, she might have worn a low dress without any disfigurement being observable. The proceeding of placing the hand behind the back in the treatment of fractured clavicle is not quite new, for M. Grout is cited by M. Malgaigne as having adopted it. M. Broca does not think this plan applicable to all cases, since it compels the patient to sleep on the opposite side; but he agrees with Malgaigne in believing that in some fractures of the clavicle, the broken ends of the bone can only be brought into apposition by placing the upper extremity in special and peculiar positions, which may be quite different in different instances.—*Journal de Medicine*, Tome xliv, 5eme Cahier.—*The Clinic.*

---

### *American Public Health Association.*

NEW YORK, September 2, 1873.

The Executive Committee of the American Public Health Association have resolved to change the time and place of the adjourned annual meeting, as designated at Cincinnati.

Instead of the meeting proposed to be held at Providence, R. I., in September, it is deemed expedient to designate the city of New York as the place, and Tuesday, the 11th of November, as the time, for the next general meeting of the Association. It is believed that this appointment will best suit the preferences of all members and friends of the Association that can attend the Autumn Meeting. These changes are rendered necessary by the pressure of official and professional duties at this season.

Please communicate to the Secretary at your earliest convenience, such information as you can give respecting papers or reports which will be prepared for that meeting, in order to enable the Executive Committee to issue in advance the synopsis of papers and topics for discussion at the meeting, as required by the Constitution.*

The opening session will begin on Tuesday forenoon of November 11th, and it is expected that important reports and discussions will occupy the attention of the Association for three or four days.

By order of the Executive Committee.

*Executive Committee.*—Stephen Smith, M.D., President; Edwin M. Snow, M.D., 1st Vice-President; John H. Rauch, M.D., Treasurer; Moreau Morris, M.D.; Henry Hartshorne, M.D.; Francis Bacon, M.D.; William Clendenin, M.D.; C. C. Cox, M.D.; John M. Woodworth, M.D.; Elisha Harris, M.D., Secretary, 301 Mott Street, New York.

* All Committees, and all members preparing scientific reports or papers to be laid before the Association, at its annual meetings, must give, in writing, the title and outline of such reports or papers to the Executive Committee, at least six weeks preceding the date of such meeting, to secure their announcement in the order of business.

### *Notice.*

A Clinic for the Treatment of Diseases of the Brain and Nervous System, will be held at Rush Medical College on every Saturday afternoon throughout the year, by Dr. W. Hay, Adjunct Professor of Theory and Practice.

